# New Serotonin-Norepinephrine Reuptake Inhibitors and Their Anesthetic and Analgesic Considerations

**DOI:** 10.3390/neurolint13040049

**Published:** 2021-10-01

**Authors:** David Fanelli, Gregory Weller, Henry Liu

**Affiliations:** Milton S. Hershey Medical Center, Department of Anesthesiology and Perioperative Medicine, Pennsylvania State University College of Medicine, 500 University Drive, Hershey, PA 17033, USA; fanellidavid@gmail.com (D.F.); gweller@pennstatehealth.psu.edu (G.W.)

**Keywords:** serotonin-norepinephrine reuptake inhibitors, serotonin, desvenlafaxine, duloxetine, levomilnacipran, milnacipran, sibutramine, serotonin syndrome

## Abstract

Serotonin-norepinephrine reuptake inhibitors (SNRIs) inhibit the presynaptic neuronal uptake of serotonin and norepinephrine and prolong the effects of the monoamines in the synaptic cleft within the central nervous system, leading to increased postsynaptic receptor activation and neuronal activities. Serotonin-norepinephrine reuptake inhibitors can have multiple clinical indications, including as the first-line agents for the management of depression and anxiety, and as analgesics in the treatment of chronic pain. The effects of reuptake inhibition of norepinephrine and serotonin are often dose-dependent and agent-dependent. There are five FDA-approved serotonin-norepinephrine reuptake inhibitors (desvenlafaxine, duloxetine, levomilnacipran, milnacipran and sibutramine) currently being marketed in the United States. As the COVID-19 pandemic significantly increased the incidence and prevalence of anxiety and depression across the country, there are significantly increased prescriptions of these medications perioperatively. Thus, anesthesiologists are more likely than ever to have patients administered with these agents and scheduled for elective or emergency surgical procedures. A thorough understanding of these commonly prescribed serotonin-norepinephrine reuptake inhibitors and their interactions with commonly utilized anesthetic agents is paramount. There are two potentially increased risks related to the continuation of SNRIs through the perioperative period: intraoperative bleeding and serotonin syndrome. SNRIs have some off-label uses, more new indications, and ever-increasing new applications in perioperative practice. This article aims to review the commonly prescribed serotonin-norepinephrine reuptake inhibitors and the current clinical evidence regarding their considerations in perioperative anesthesia and analgesia.

## 1. Introduction

Serotonin-norepinephrine reuptake inhibitors (SNRIs) are among the most commonly prescribed medications in the United States annually [1]. As their name suggests, the principle mechanism of action is the inhibition of presynaptic neuronal uptake of 5-HT (serotonin) and norepinephrine following release from the synaptic cleft. Prevention of reuptake prolongs the persistence of these monoamines in the synaptic cleft within the central nervous system (CNS). Accordingly, this results in increased postsynaptic receptor stimulation and additional post synaptic neuronal transmission. There are five principal SNRIs that are currently approved by the Food and Drug Administration (FDA) for use in the United States (Table 1) [2,3,4,5,6,7,8]. SNRIs are often considered to have a ‘dual action’ on account of their mechanism, however the specific degree of reuptake inhibition of norepinephrine and serotonin is both dose- and agent-dependent.

SNRIs are a versatile class of medications with a wide variety of clinical applications [9]. Specific clinical applications vary between SNRIs; however, these drugs are commonly prescribed as first-line agents for the treatment of depression, anxiety and fibromyalgia, among other medical conditions [1,9]. Additionally, there are emerging applications for their use in the treatment of chronic pain. Anxiety and depression rates have been rising nationally, and this trend has been exacerbated by the COVID-19 pandemic, which significantly increased the incidence of these diseases [10,11,12]. Accordingly, anesthesiologists are more likely than ever to encounter patients who take these agents and present for elective or emergency surgical procedures. The concerns over continuing SNRIs through the perioperative period are mainly related to their theoretically increased risks of intraoperative bleeding and the development of serotonin syndrome. A thorough knowledge of commonly prescribed SNRIs and their interactions with commonly utilized anesthetic agents is critically important. This paper aims to review commonly prescribed SNRIs and current evidence regarding their considerations in anesthesia.

## 2. Serotonin-Norepinephrine Reuptake Inhibitors

### 2.1. Desvenlafaxine

Approved by the Food and Drug Administration (FDA) in 2008, desvenlafaxine is a potent SNRI that potentiates neurotransmitter activity of serotonin and norepinephrine within the CNS. The drug is the synthetic form of the SNRI venlafaxine’s major active metabolite, and is principally used in the treatment of major depressive disorder (MDD). Desvenlafaxine is renally eliminated, but first undergoes hepatic conjugation with minor metabolism through the CYP3A4 pathway. Current evidence suggests equal efficacy between desvenlafaxine and venlafaxine in treatment of MDD [13]. However, desvenlafaxine may be a better drug selection in patients with decreased P450 CYP2D6 activity (crucial in the metabolism of venlafaxine and many other medications), who may be poor metabolizers [14]. Overall, desvenlafaxine has a favorable side effect profile. However, there are several key theoretical concerns for anesthesiologists, including the development of hypertension, angioedema and serotonin syndrome. At least one case report implicated desvenlafaxine in the development of refractory intraoperative hypertension [15]. Additionally, there is anecdotal evidence regarding the development of Takotsubo Cardiomyopathy with resultant intraoperative cardiac arrest, although this appears rare [16]. Specific guidelines for the perioperative management of desvenlafaxine apart from recommendations for SNRIs as a class are lacking. A 14-day tapering process is recommended if the decision is made to discontinue the medication in the perioperative period [13,15].

### 2.2. Duloxetine

Duloxetine is amongst the mostly commonly prescribed drugs annually in the United States. Accordingly, anesthesiologists are likely to encounter patients taking this drug, which was approved by the FDA in 2004 and is currently the first-line therapy for a variety of psychiatric diagnoses including MDD, generalized anxiety disorder, fibromyalgia, diabetic peripheral neuropathy and musculoskeletal pain. Additionally, duloxetine has a number of off-label uses, including treatment of peripheral neuropathy secondary to chemotherapy administration and urinary incontinence [17]. Like many other SNRIs, metabolism is through the CYP450 family of enzymes, specifically through the CYP2D6 and CYP2A2 pathways via oxidation and conjugation. Accordingly, interpatient variations in enzyme activity may contribute to varied clinical effects [18]. Duloxetine has an established safety profile and is generally well-tolerated. However, there are a number of significant severe adverse reactions including hypertensive crisis, Stevens-Johnson syndrome, serotonin syndrome, and fulminant liver failure that are of particular concern for anesthesiologists [19]. Recent research of duloxetine is largely centered on applications as an adjunct therapy in chronic pain management [20,21,22,23]. There is also an expanding role for duloxetine as an adjunct therapy in the acute postoperative period as part of a multimodal analgesia regimen, although results are mixed [24,25,26]. Trials supporting its efficacy are mostly centering on opioid-sparing effects with concurrent therapy [27]. More evidence is likely necessary to definitively conclude that antidepressants have a distinct role in the management of postoperative pain, with better characterization of the risk benefit ratio [28]. An additional consideration regarding duloxetine for anesthesiologists is the interaction of the drug with electroconvulsive therapy. Although largely continued throughout electroconvulsive therapy without an issue, an isolated case occurred where a duloxetine-lithium combination therapy precipitated ventricular tachycardia during an electroconvulsive therapy session [29]. Duloxetine can likely be continued through the perioperative period; however, if discontinuation is required, a 14-day taper is suggested [30]. 

### 2.3. Levomilnacipran

Levomilnacipran is the most recently FDA-approved SNRI (2013), and is currently indicated for the treatment of MDD. However, additional applications for levomilnacipran are currently under investigation [31]. The drug is principally metabolized by CYP3A4 via desethylation and hydroxylation with further conjugation. Levomilnacipran differs from other SNRIs because it has a doubled potency of norepinephrine reuptake inhibition compared to serotonin [32]. Specific studies evaluating levomilnacipran in the perioperative, intraoperative, and postoperative settings are currently lacking. The more recent FDA approval and relatively lower number of prescriptions may be contributing factors to an overall low volume of evidence. Furthermore, levomilnacipran has not yet been investigated for off-label uses, unlike many other SNRIs. Levomilnacipran does have a number of side effects that are relevant to anesthesiologists, including hypertension and serotonin syndrome. As previously discussed, these adverse reactions are common to the SNRI class of medications. Recommendations on the continuation of levomilnacipran in the perioperative period are lacking, but likely mirror other SNRIs.

### 2.4. Milnacipran

Milnacipran is an older SNRI that obtained FDA approval for use in the treatment of fibromyalgia in 1996, and is the first-line treatment for this indication. An additional off-label use includes the treatment of MDD, and is often a second-line therapy for this use [33]. Current research into milnacipran is exploring its role in the treatment of neuropathic pain, although early results are not promising [34]. Of note, unlike many other SNRIs, milnacipran does not undergo metabolism via the CYP450 system. The side effect profile is similar to other SNRIs, with the most severe effects being hypertensive crisis, erythema multiforme, Stevens-Johnson syndrome, fulminant hepatitis, and serotonin syndrome. Evidence related specifically to anesthesia, outside of general concerns regarding the SNRI class, are lacking. A 14-day taper is recommended if the decision is made to discontinue in the perioperative period.

### 2.5. Sibutramine

Sibutramine is an SNRI that prevents dopamine reuptake, in addition to blocking the reuptake of serotonin and norepinephrine. Sibutramine reduces the reuptake of norepinephrine (by 73%), serotonin (by 54%), and dopamine (by 16%). The drug was initially approved for use in 1998 as an appetite suppressant for the treatment of obesity. However, it was voluntarily withdrawn from the US markets in 2010. Studies were suggestive of an increased risk of cardiovascular adverse events including non-fatal stroke and non-fatal heart attack [35]. Despite this risk profile, sibutramine is still available in countries outside of the United States. Unlike many other drugs in the SNRI class, there is no utility as an antidepressant, despite its initial evidence of efficacy [36]. 

### 2.6. Tramadol

Tramadol was patented in the 1960s but was not approved by the FDA until 1995. The drug’s principle mechanism of action is through opioid µ-receptor agonism; however, the drug also functions as a serotonin and norepinephrine reuptake inhibitor. Although not a traditional SNRI, tramadol’s partial SNRI mechanism of action merits consideration in this paper. Accordingly, tramadol’s principle mechanism of action as a µ-receptor agonist precludes its use as a first-line antidepressant, unlike most other drugs in the SNRI class. Instead, tramadol is FDA-approved for the management of both acute and chronic pain.

Due to its unique mechanism of action and prolonged time on the market, tramadol has been extensively investigated for off-label uses, more so than other drugs with an SNRI mechanism of action. These include treatment of cancer pain, adjunct therapy for peripheral nerve blocks, and neuraxial anesthesia. Additionally, tramadol has also been extensively evaluated for the treatment of neuropathic pain. However, a recent meta-analysis suggested only modest benefit [37]. The combined mechanisms are thought to be more effectively modulate to the transmission of pain [38]. Tramadol is metabolized through the CYP450 family of enzymes (mostly CYP2D6 and CYP3A4) via conjugation, N- and O-demethylation, and glucuronidation or sulfation. Although well-tolerated, adverse effects of tramadol include respiratory depression, physical dependence, seizures and serotonin syndrome. Risk of serotonin syndrome and seizures are unique to tramadol amongst other opioids, and is attributed to its serotonin-norepinephrine mechanism of action [39,40]. 

Tramadol is more widely utilized in the preoperative, intraoperative, and postoperative period compared to other medications with serotonin-norepinephrine reuptake pharmacodynamics. Intraoperative use of tramadol is limited in favor of other pure opioid agonists; however, tramadol may be administered in the perioperative period as part of an effective multimodal pain regimen [38]. Tramadol has also been utilized as an adjunct to local anesthetic for a neuraxial blockade via an intrathecal approach, epidural, or combined spinal-epidural with varying success [41,42,43]. Adjunct agents are a method to prolong the duration of the blockade while minimizing unwanted side effects. These blocks are commonly employed by anesthesiologists for a variety of surgeries.

In addition to applications as an adjunct to neuraxial anesthesia, tramadol has been utilized in combination with local anesthetics for applications in regional anesthesia. While tramadol does not have efficacy as a solo agent for regional anesthesia, it may improve the efficacy of local anesthetics [44]. This has been found regarding upper extremity blocks in particular [45]. However, not all data is supportive of tramadol as an effective adjunct for regional anesthesia [46]. There is some evidence suggestive of tramadol as a solo effective local anesthetic for maxillary infiltration [47]. The concurrent use of tramadol with other antidepressants is of specific interest due to the variety of applications of tramadol in the perioperative period and theoretical concerns over the development of serotonin syndrome. Current evidence suggests tramadol use with monoamine oxidase inhibitors should be avoided, but concurrent use with other antidepressants is not contraindicated [48]. 

Applications of tramadol in the postoperative period are related to its successful prevention of postoperative shivering [49,50,51]. Additionally, this benefit appears following a variety of different anesthetics, including remifentanil-induced shivering as well as following subarachnoid blocks [52,53]. Tramadol continues to draw interest as one component of a multimodal postoperative pain management strategy [54]. 

### 2.7. Venlafaxine

Venlafaxine is amongst the most widely used drugs of the SNRI class, despite only obtaining FDA-approval in 2005. It is currently approved in the treatment of generalized anxiety disorder, MDD, social phobia, and panic disorder. Additionally, venlafaxine is utilized off-label for the treatment of attention deficit hyperactive disorder, bipolar disorder, diabetes-induced neuropathy, obsessive compulsive disorder, peripheral neuropathy due to chemotherapy, post-traumatic stress disorder, and tension headache prophylaxis. In addition to reducing serotonin and norepinephrine reuptake, venlafaxine also inhibits the reuptake of dopamine. Venlafaxine is metabolized by P450 CYP2D6, and is subject to variations in metabolism related to enzyme activity levels. Overall, the drug has a favorable side effect profile; however, patients should be closely monitored for serotonin syndrome amongst other issues which may be encountered in the pre-, intra-, or postoperative setting [55]. Additionally, there is at least one case of opioid-induced rigidity precipitated by venlafaxine [56]. More recently, venlafaxine has been evaluated for the treatment of chronic neuropathic pain [22,57,58]. Another area of active research is investigating venlafaxine in the prevention of the development of chronic pain following surgery [59]. 

## 3. Discussions

### 3.1. Preoperative Considerations

Current guidelines regarding the continuation of SNRIs in the perioperative period remain incomplete; however, patients are generally encouraged to continue taking their medication as prescribed [60]. Our current institutional practice is to continue SNRIs through the day of surgery after evaluating the patient as part of a comprehensive perioperative assessment. As leaders in perioperative medicine, it is critical for anesthesiologists to appropriately counsel patients on the continuation of these medications. The concerns over the continuation of SNRIs through the perioperative period are largely related to a theoretically increased risk of intraoperative bleeding and the risk of developing serotonin syndrome, as discussed below [61]. These risks must be weighed against the clinical consequences of discontinuing an SNRI. Patients dependent on these medications may experience an exacerbation of psychiatric symptoms, which carry risks, especially when dealing with psychiatric medications. Furthermore, SNRIs require a 14-day taper to safely stop the medication prior to surgery [13,15,60]. Optimal timing on restarting these medications is also poorly defined. Accordingly, patients should be assessed on an individual basis.

### 3.2. Intraoperative Considerations

SNRIs are not commonly administered as part of the routine maintenance of anesthesia. However, anesthesiologists may encounter the effects of these medications intraoperatively. In one study, Kimura et al. found that patients taking SNRIs were more likely to experience refractory hypotension as compared to treatment by phenylephrine and ephedrine, although these patients responded appropriately to dobutamine or norepinephrine [62]. The group speculated that sympathetic activity may be affected by the interaction of SNRIs and anesthetics in some patients. The effects of SNRIs have also been investigated as related to the transfusion requirements for patients undergoing cardiac surgery. Serotonin reuptake is known to effect platelet aggravation, and evidence suggests that significant serotonin reuptake is a risk for spontaneous bleeding [61,63]. This risk may be exacerbated with concurrent nonsteroidal anti-inflammatory use [61,64]. However, Smith et al. found that SNRI/SSRI use was not associated with significantly increased transfusion or risk of bleeding during cardiac surgery. The group concluded that holding SNRI/SSRIs prior to cardiac surgery is unnecessary as it relates to the risk of perioperative bleeding [63]. In contrast, Mawardi et al. found that SNRI use independently increases risk of gastrointestinal bleeding in LVAD patients [65]. Recent research also suggests that SSRIs/SNRIs may be associated with poor postoperative outcomes following hepatic resection, and should be held in the perioperative period [66]. 

Apart from the risk of intraoperative hemorrhage and hypotension, anesthesiologists must be vigilant for the development of serotonin syndrome, discussed further below. This is a feared sequela of SNRIs, and must be managed in a timely and appropriate manner to prevent adverse outcomes.

### 3.3. Postoperative Considerations

The principle considerations of SNRIs in the acute postoperative period are related to their efficacy, as part of a multimodal postoperative pain strategy and as a risk factor for the development of serotonin. Multimodal analgesia has emerged as a central component of pain management following surgery [54,67]. Additionally, this care practice is a critical component in enhanced recovery after surgery (ERAS) protocols. These protocols are designed to address the comprehensive surgical experience, which includes postoperative pain management [68]. Multimodal analgesia “involves the use of multiple, simultaneous mechanisms of pain control acting synergistically to improve analgesic effect and reduce the doses of any single agent to minimize risks of side-effects” [69]. SNRIs have potential applications in this space. For example, SNRIs were recently evaluated for recovery following total knee arthroplasty with favorable results [70]. 

Commonly prescribed SNRIs are summarized in Table 1.

### 3.4. Serotonin Syndrome

Serotonin syndrome is a collection of symptoms that develops following the administration of some serotonergic drugs, including SNRIs. In addition to SNRIs, a number of other medications and drugs can trigger the development of serotonin syndrome (Table 2). Furthermore, many of these medications are routinely encountered by anesthesiologists in the perioperative setting. As the number of patients who take SNRIs continues to increase, the number of cases of serotonin syndrome can reasonably be expected to increase as well [71,72]. 

Most cases of serotonin syndrome develop following administration of two agents that both cause serotonergic release [73]. The syndrome typically presents as a clinical triad, including behavioral/cognitive changes combined with neuromuscular and neurovegetative features [74]. One of the challenges of diagnosing and managing serotonin syndrome lies in the variability of the classic triad of symptoms. Mild cases may present tachycardia, myoclonus, and sweating [72,74]. More severe cases are characterized by the development of hyperthermia and hypertension [71]. The most threatening cases may produce temperatures in excess of 41.1 °C, combined with severe hypertension that may ultimately lead to shock and death [72]. 

Recognizing and treating serotonin syndrome is a significant challenge, in part because of a broad differential which includes anticholinergic toxicity, meningitis, neuroleptic malignant syndrome, malignant hyperthermia, encephalitis and sedative-hypnotic withdrawal, amongst others. This differential is particularly challenging for anesthesiologists as many of these conditions may be encountered during or following general anesthesia.

Serotonin syndrome remains a clinical diagnosis, and lacks specific diagnostic testing at this time. Accordingly, a number of screening guidelines have been developed to assist with the diagnosis. The Hunter Toxicity Criteria Decision Rules is the most commonly utilized and best-validated tool to aid in this process, although this remains controversial [75,76]. The criteria require that a patient must have taken a serotonergic agent, and must also meet one of the following conditions: (1) spontaneous clonus; (2) inducible clonus plus diaphoresis or agitation, (3) ocular clonus plus diaphoresis or agitation; (4) tremor plus hyperreflexia; or (5) hypertonia plus a temperature above 38 °C plus inducible clonus or ocular clonus [75]. The triggering factors and their mechanisms are summarized in Table 2.

One of the challenges in the prevention, diagnosis, and treatment of serotonin syndrome is the variety of medications that may precipitate a crisis. Furthermore, serotonin syndrome can be precipitated at any point during the preoperative, intraoperative, or postoperative period. Therefore, constant vigilance is required. In the perioperative period, serotonin syndrome may manifest in atypical ways, and should be considered with any abnormal response to preoperative medications [55,77,78]. Many times, toxicity is related to other medications that patients may be taking on the day of surgery, including nutritional or herbal supplements [79]. 

Serotonin syndrome may also present intraoperatively, and may be due to interactions with a variety of agents routinely administered during anesthetics, such as methylene blue [80,81]. An abnormal response to anesthesia, including movement despite adequate depth of anesthesia, may be a sign of serotonin syndrome [82]. A delayed emergence in the setting of risk factors may be attributed to serotonin syndrome [83,84,85]. Malignant hyperthermia is a condition uniquely encountered by anesthesiologists with many symptoms that are similar to those of serotonin syndrome. Unlike serotonin syndrome, malignant hyperthermia is triggered by inhalational volatile anesthetics (halothane, enflurane, isoflurane, sevoflurane, desflurane) and succinylcholine. Following exposure, susceptible individuals typically present with hypercarbia, tachypnea, and tachycardia as early manifestations. Hyperthermia, generalized rigidity, and peaked T waves secondary to hyperkalemia are delayed and inconsistent findings. The late clinical sequalae of malignant hyperthermia can produce Coca-Cola colored urine (myoglobinuria) and disseminated intravascular coagulation. Under anesthesia, there are similarities to serotonin syndrome that may be difficult to distinguish; however, doing so is critically important as treatment is unique to each condition. Malignant hyperthermia is managed acutely with the discontinuation of the triggering agents, followed by administration of dantrolene with a loading dose of 2.5 mg/kg, followed by an infusion of 1 mg/kg until the symptoms begin to abate.

Serotonin syndrome may also present postoperatively where the differential diagnosis also includes postoperative delirium, residual neuromuscular blockade and delayed onset malignant hyperthermia. As previously discussed, differentiating between these diagnoses is important as management varies. Another challenge is that serotonin syndrome following surgery may have a delayed presentation, complicating diagnosis [19]. 

Due to the nature of serotonin syndrome, the breadth of the precipitating agents, and the relative rarity of the event, most research in surrounding the administration of anesthesia is based on case reports. Larger multi-patient studies evaluating this syndrome are lacking. The treatment of serotonin syndrome is principally based on supportive care. This involves the discontinuation of serotonergic agents, normalization of hemodynamics, treatment of hyperthermia, benzodiazepines for sedation, and serotonin antagonists such as cyproheptadine. Cyproheptadine functions as a histamine-1 receptor antagonist that also has 5-HT1A and 5-HT2A antagonistic properties combined with anticholinergic activity [86]. With early recognition of symptoms and treatment, most patients recover within 24 h of the discontinuation of the offending agent [87]. Prognosis is generally favorable. Genetic testing has been utilized to evaluate patients following serotonin syndrome to assess abnormal drug metabolism [88]. 

### 3.5. Emerging Applications

As previously alluded to, SNRIs continue to gain further acceptance as first-line agents for the treatment of a variety of conditions. Furthermore, SNRIs are under investigation for new and emerging applications. This is most evident in their use for the treatment of chronic pain conditions, with a particular emphasis on the use of SNRIs in the treatment of neuropathic pain [89,90,91,92]. In particular, duloxetine, as previously discussed, has emerged at the forefront of agents utilized in the treatment of this condition. Other agents have been investigated in the treatment of neuropathic pain as well, including venlafaxine and desvenlafaxine, although clinical significance of efficacy is lacking [93,94]. Research suggests that duloxetine may be useful in the treatment of chronic low back pain amongst other conditions [95,96]. SNRIs have also been investigated in the treatment of non-traditional chronic pain syndromes such as chronic central post-stroke pain [97]. Outside of chronic pain, there is an interest in the use of SNRIs—apart from milnacipran—in the treatment of conditions such as fibromyalgia, although results have been mixed [98]. SNRIs as a prophylactic migraine therapy has also been an area of active research, however current results are inconclusive [99,100,101]. 

The SNRI duloxetine has also been investigated as an adjunct therapy in the treatment of osteoarthritis with modest results [102]. While osteoarthritis is traditionally considered a peripheral pain syndrome, a subset of patients may have a centralized pain component responsive to monoamine modulating agents, amongst others [103]. SNRIs have also been utilized in the treatment of cancer-related pain [104]. Additionally, this benefit may extend beyond pain reduction. Tramadol has recently been found to contribute to enhanced outcomes in patients undergoing treatment for breast cancer [105]. Other SNRIs have been investigated for their effect on outcomes in cancer treatment as well although data is inconclusive [106]. 

## 4. Conclusions

SNRIs are a commonly utilized class of medications with an ever-increasing variety of applications. All of the SNRIs discussed above are likely to be encountered by anesthesiologists in perioperative settings. They have a variable side effect profile relative to their clinical benefits, and they are generally well-tolerated. The recognition of serotonin syndrome and the multitude of triggering agents commonly utilized by anesthesiologists is of critical importance. Emerging applications in chronic pain and use within multimodal analgesia protocols can reasonably be expected to augment use. Tramadol as a mixed opioid agonist with a combined SNRI mechanism of action has a variety of applications in the perioperative, intraoperative, and postoperative space. Large randomized trials evaluating the role of specific interactions of SNRIs with anesthetic agents are lacking, but are an area of active research. Anesthesiologists, as perioperative experts, must have an in-depth knowledge of SNRIs, their common applications, and the implications for anesthetics.

## Figures and Tables

**Table 1:** Commonly-prescribed serotonin-norepinephrine reuptake inhibitors.

Drug (FDA Approval)	FDA Approved Indications for Use	Bioavailability (Oral)	Protein Binding	Metabolism	Elimination	T_1/2_ (Hours)	Severe Side Effects
Desvenlafaxine (2008)	Major Depressive Disorder	80%	30%	Hepatic; conjugation, primary; CYP3A4, minor pathway	Renal: 45%, unchanged; up to 24% changed	10–11.1	Hypertension, angioedema, suicidal ideation, serotonin syndrome
Duloxetine (2004)	Major Depressive Disorder, Generalized Anxiety Disorder, Fibromyalgia, Diabetic Peripheral Neuropathy, Musculoskeletal pain	30–80%	>90%	Hepatic: P450 CYP2D6 and CYP1A2 via conjugation and oxidation	Fecal: 20% Renal: 70% as metabolites	12	Hypertensive crisis, Steven-Johnson Syndrome, withdrawal syndrome, serotonin syndrome, liver failure
Levomilnacipran (2013)	Major Depressive Disorder	92%	22%	Desethylation by CYP3A4, and hydroxylation with further conjugation	Renal: 58% unchanged, 27% identifiable metabolites	12	Hypertension, suicidal ideation, serotonin syndrome, drug withdrawal, seizure
Milnacipran (1996)	Fibromyalgia	85–90%	13%	Hepatic	Renal: 50% to 60% unchanged drug	6–8	Hypertensive crisis, Erythema multiforme, Stevens-Johnson syndrome, Fulminant hepatitis, suicidal ideation, serotonin syndrome
Tramadol (1977)	Pain management	70–75%	20%	Hepatic: extensive via CYP2D6 and CYP3A4, conjugation, N- and O-demethylation, and glucuronidation or sulfation	Renal excretion: 60% as metabolite; approximately 30% unchanged	5.6–6.7	Dyspnea, respiratory depression, serotonin syndrome
Venlafaxine (1994)	Panic disorder, generalized anxiety disorder, major depressive disorder, social phobia	42%	27–30%	Hepatic: extensive first-pass via P450 CYP2D6	Fecal: 2% Renal: 87%, 82% as metabolites, 5% unchanged	5	Neuroleptic malignant syndrome, Serotonin syndrome, suicidal ideation

**Table 2:** Triggering agents of serotonin syndrome.

Medication or Medication Class	Mechanism of Serotonin Modulation
Cocaine, meperidine, tramadol, cyclobenzaprine, sibutramine, dextromethorphan, selective serotonin reuptake inhibitors, St. John’s wort, cyclobenzaprine, 5-HT3 receptor antagonists, serotonin norepinephrine reuptake inhibitors, trazadone, cyclic antidepressants	Inhibits presynaptic neuronal reuptake of serotonin
Fentanyl, lasmiditan, lysergic acid diethylamide, triptans, ergot derivatives, metaxalone	Functions as a direct agonist at serotonin receptor
Monoamine Oxidase Inhibitors (MOAIs), MOAI-A inhibitors, MOAI-B inhibitors	Inhibits metabolism of serotonin through inhibition of monoamine oxidase

## Data Availability

Not applicable.
